# Blood Pressure Control, Accessibility, and Adherence to Antihypertensive Medications: Patients Seeking Care in Two Hospitals in the Ashanti Region of Ghana

**DOI:** 10.1155/2021/9637760

**Published:** 2021-07-15

**Authors:** Nana Ofori Adomako, Afia Frimpomaa Asare Marfo, Mercy Naa Aduele Opare-Addo, Nathaniel Nyamekye, Frances Thelma Owusu-Daaku

**Affiliations:** Department of Pharmacy Practice, Faculty of Pharmacy and Pharmaceutical Sciences, Kwame Nkrumah University of Science and Technology, Kumasi, Ghana

## Abstract

Hypertension is the second leading cause of death in Ghana, partly accounting for two-thirds of all medical admissions and more than 50% of deaths. This study aimed to comparatively evaluate adherence and accessibility to antihypertensive medications at two different levels of healthcare facilities in Kumasi, Ghana, and determine factors associated with medicine accessibility and adherence. A cross-sectional study involving outpatient department (OPD) hypertensive patients, 143 at KNUST Hospital (UHS) and 342 at Komfo Anokye Teaching Hospital (KATH), was conducted using a semistructured questionnaire. Correlations were drawn to evaluate the effect of accessibility and adherence on blood pressure control. A face-to-face interview was also conducted with relevant stakeholders involved in procurement of medicines. Blood pressure was uncontrolled in 50.4% (*n* = 72) of participants at UHS and 52.9% (*n* = 181) at KATH. With respect to medicine accessibility, 98.8% (*n* = 338) and 42.9% (*n* = 61) received at least one medication from the hospital pharmacy of KATH and UHS, respectively. Using MARS-10, 49.2% (*n* = 70) and 52.9% (*n* = 181) were nonadherent in UHS and KATH, respectively. There was a significant association between adherence and BP control at both UHS (*p*=0.038) and KATH (*p*=0.043). At UHS, there was a significant association between accessibility to medicines at the hospital and BP control (*p*=0.031), whilst at KATH, no significant association was observed (*p*=0.198). Supply chain practices and delays in payment by the NHIA affected accessibility to antihypertensive medications. Blood pressure control was inadequate among participants in both facilities. Accessibility to medicines was better at the tertiary facility compared to the secondary facility. Increased accessibility and adherence to antihypertensives were related to blood pressure control in both facilities. Good supply chain practices and prompt payment by the National Health Insurance Authority would enhance accessibility to antihypertensive medications.

## 1. Introduction

The incidence of hypertension and its prevalence and death due to complications have increased globally. The prevalence of hypertension in sub-Saharan Africa is estimated to be 38% in some communities with an expected rise in numbers by 2025 [1]. Population-based studies conducted in Ghana have shown that the prevalence of hypertension ranges from 19 to 48% [2]. This has had a significant impact on the occurrence of stroke as a comorbidity and resultant mortality [3]. The increase in burden of hypertension may be linked to high population growth mostly in the urban regions of Ghana and lifestyle factors including abusive use of alcohol, poor diet, and lack of exercise [4]. Obesity and increase in waist-to-hip circumference are equally important contributory factors [4]. Despite this, the awareness, treatment, and control of hypertension continue to be low [5].

Poor adherence is the most important cause of uncontrolled BP. Research has shown that an estimate of about 50–70% of hypertensive patients do not adhere to their antihypertensive therapy as prescribed [6]. This results in unnecessary overprescription of drugs, deterioration of disease condition, increase in hospitalization as well as length of stay with resultant reduction in optimal clinical benefits, and increase in risk of cardiovascular events [7]. Nonadherence is an important cause of failure to achieve target BP levels in about sixty percent of all hypertensives [8]. Consequences of nonadherence impact significantly on healthcare costs [9].

The Ghana National Health Insurance Scheme (NHIS) was instituted as a social intervention to reduce cost and to increase accessibility to healthcare. However, due to setbacks resulting from delays in payment of service providers and lack of financial sustainability, supply of medicines to public health facilities has been a challenge. In addition, uncoordinated care, inadequate funds, shortage of healthcare professionals, and low health insurance coverage contribute to the weak healthcare system in Ghana [10]. Even though some levels of hypertension management are covered by the NHIS, some patients may not benefit from the insurance coverage due to these setbacks [11]. Thus, inadequate supply of antihypertensives could lead to poor blood pressure control and nonadherence. In Ghana, data on accessibility to antihypertensive medication and its effect on blood pressure control are limited.

This study therefore sought to investigate and compare the accessibility of antihypertensive medication and its effect on adherence and blood pressure control at two different levels of healthcare facilities. Komfo Anokye Teaching Hospital (KATH) is a 1200-bed tertiary and referral hospital easily accessible to people in the Kumasi and the northern sector of Ghana [12]. University Health Services (UHS), on the contrary, is a 125-bed secondary hospital offering healthcare services to the university community and surrounding suburbs including Kotei, Deduako, Bomso, and Ayigya [13]. The diversity in services rendered and differences in levels of healthcare make these facilities suitable for the study.

Data from this study will enable relevant stakeholders of healthcare appreciate the factors that affect accessibility to medicines and its effect on adherence and blood pressure control in order to institute measures to achieve holistic outcome-oriented patient care.

## 2. Materials and Methods

### 2.1. Study Design and Setting

A hospital-based cross-sectional study was carried out during the hypertensive clinic days at University Health Services (UHS) and Komfo Anokye Teaching Hospital (KATH) in Kumasi.

### 2.2. Sampling and Sample Size

Participants enrolled for the study were hypertensive patients aged between 25 and 80 years visiting UHS or KATH for review. Their oral consent was sought prior to the study. In estimating the minimum sample size, Glenn's formula was used (no. = (*Z*^2^*PQ*) ÷ *E*^2^, where *Z* is the confidence interval, *P* is the prevalence rate, *Q *= (1 − *P*), and *E* is the desired level of precision. The confidence interval was set at 95% with a precision of ±5. The power of the study was estimated to be 91.5% using SPSS version 25. A sample size of 408 was obtained. To accommodate a 10% margin of error, a stronger statistical power, and effect size, a total of 485 participants were interviewed for the study, 342 from KATH and 143 from UHS, in proportion to estimated daily visits at these healthcare facilities. We also interviewed five pharmacists: the director of pharmacy (DP), deputy director (DDPS), two clinical pharmacists (CP), and head of the medical store (MS). These individuals were directly responsible for the acquisition and supply of medicines in both facilities.

### 2.3. Inclusion and Exclusion Criteria

All patients diagnosed with hypertension and on medications who were willing to participate in the study were included. Pregnant women and newly diagnosed patients who were yet to be put on medications were excluded.

### 2.4. Development and Validation of the Questionnaire

In assessing access to medicines, indicators were adapted from the WHO level 1 indicators on access and adherence to drugs by patients [14]. Level of adherence was assessed using Medication Adherence Rating Scale-10 [15]. A set of questions were adapted from the WHO Developing Integrated Response of Health Care Systems to Rapid Population Ageing Questionnaire for Hypertensive Patients to assess patient knowledge and the counselling received [16]. A qualitative questionnaire was adapted from the WHO standard DTC handbook [17] and the USAID supply chain KPI reference to assess stakeholders' commitment in making medicines readily available [18]. A copy of the questionnaire has been included as an appendix (Supplementary Materials (available here)).

### 2.5. Data Collection

The questionnaires, which sought to obtain information on patient demographics, accessibility, and adherence to medications, were distributed by PharmD postgraduate students trained in survey studies, specifically in areas of obtaining informed consent, the need to address terms of confidentiality, how to explain the interview format, questionnaire completion, and overall implementation of the study protocol. The questionnaires were read, explained, and translated into the local dialect to the participants as needed. Participants were taken through the questionnaires, and the sheets were collected after completion of the interview.

Data collected were demographic characteristics, duration of condition, blood pressure measurements, antihypertensive medications prescribed, and antihypertensives dispensed. The blood pressure was obtained from the patients' folder which was a triplicate measurement with an average of the second and third systolic and diastolic readings taken within a constant time interval of five minutes [19]. Regarding accessibility, factors assessed included participants' travel time when accessing hospitals, means of transport as well as affordability, and mode of payment for healthcare services rendered. They were also asked about the medications they were currently taking and compared to what was stated in the patient folder in order to assess their knowledge of their medicines, regimen, and the medicine counselling received during dispensing. In assessing adherence, the Medication Adherence Rating Scale-10 (MARS-10) was used where patients who scored between 0 and 5 had low adherence and 6 and 10 had high adherence.

Questionnaires were filled anonymously. In order to prevent bias, the interviewers followed a standardized pattern in translating and explaining the content of the questionnaires to respondents. An interview guide was used to interview stakeholders in the supply of medicines at the facility. A semistructured qualitative interview guide was employed as a data collection tool. This gives participants the flexibility to express their unrestricted views. Interviews with stakeholders on accessibility were audiotaped, transcribed, and analysed for thematic content.

### 2.6. Statistical Analysis

Data obtained were analysed using IBM-Statistical Package and Service Solutions (IBM-SPSS) version 25 tool for data and multinomial logistics regression (MLRM) using R packages. Normality was tested using Shapiro–Wilk test. Significant association between variables was assessed with the chi-square test. Statistical significance was set at *p* < 0.05 with a confidence interval of 95%. Accessibility to antihypertensives, adherence, and BP control in the two levels of health were compared.

### 2.7. Ethical Clearance

All procedures were in compliance with ethical standards obtained from the Committee for Human Research Publication of KNUST (CHRPE/AP/410/18), and informed consent was sought from all participants. The study was reported in accordance with the preferred standard in reporting observational studies in epidemiology (STROBE) [20].

## 3. Results

### 3.1. Demographic and Clinical Characteristics

Of the 143 participants enrolled from UHS, the mean age was 60.87 ± 10.76. There were more females (66.2% (*n* = 94)) than males. At KATH, the mean age was 60.16 ± 11.50 with a slight majority falling between the ages of 61 and 70 years, representing 54.4% (*n* = 186). Similar to UHS, more females (72.5% (*n* = 248)) than males were enrolled. Diabetes mellitus was the commonest comorbid condition at UHS with stroke being the commonest at KATH. There was a normal distribution which was seen for all variables (Table 1).

### 3.2. Accessibility to Healthcare Facilities

The mean travel time in minutes to the health facilities was 25.29 ± 14.72 and 53.29 ± 17.86 for UHS and KATH, respectively. The commonest mode of transport for both UHS (76.2% (*n* = 109)) and KATH (76.2% (*n* = 245)) was public transport. There was a statistically significant association between time taken to access facility and adherence in both UHS (*p*=0.033) and KATH (*p*=0.006) A negatively skewed distribution was observed for time taken to reach the facility and mode of transport (Table 2).

### 3.3. Insurance Status of Participants

At UHS, 86.7% (*n* = 124) were covered by the NHIS, whilst 86.5% (*n* = 296) were covered at KATH. A representation of 8.4% (*n* = 12) and 0.3% (*n* = 1) utilized private insurance companies at UHS and KATH, respectively. A negatively skewed distribution was seen for insurance coverage (Table 3).

### 3.4. Antihypertensive Medications Prescribed for Participants

At UHS, 31.4% (*n* = 45) were given a combination of calcium channel blocker with an angiotensin-converting enzyme inhibitor or angiotensin receptor blocker and diuretics, while at KATH, the commonest combination was calcium channel blocker with an angiotensin-converting enzyme inhibitor or angiotensin receptor blocker and diuretics, representing 38.2% (*n* = 131). Other common antihypertensives administered in addition to the combinations included methyldopa, hydralazine, and beta blockers (Table 4).

### 3.5. Awareness and Adherence to Antihypertensive Medications

Of the 143 participants at UHS, 59.4% (*n* = 85) could not recall precautionary measures and counselling given; 49.2% (*n* = 70) were nonadherent. At KATH, 72.5% (*n* = 248) could not recall precautionary measures; 52.9% (*n* = 181) were nonadherent. There was a statistically significant association between awareness of precautionary measures and counselling and BP control at both UHS (*p*=0.023) and KATH (*p* < 0.001) as well as adherence and BP control at UHS (*p*=0.038) and KATH (*p* = 0.043) (Table 5).

### 3.6. Accessibility to Antihypertensive Agents

Out of the 143 participants at UHS, 50.8% (*n* = 73) received 70% and above of their prescribed antihypertensives from the hospital pharmacy, whilst that of KATH was 35.1% (*n* = 120) out of 342 participants. There was a significant association between accessibility to medicines at UHS and BP control (*p*=0.031). There was no significant relationship between accessibility of antihypertensives and BP control at KATH (*p*=0.198) (Table 6).

### 3.7. Stakeholder's Interview

From the interview, it was deduced that availability of medicines was dependent on the Drug and Therapeutics Committee who were tasked with the development of hospital formularies, preparation of treatment guidelines, and ensuring the sustained availability of medicines in the healthcare facilities. The interviews sought to identify the role of supply chain and NHIS processes as determinants of accessibility to medicines.

### 3.8. Accessibility of Medicines: The Role of the Supply Chain in UHS and KATH

Medicines are supplied to both hospitals via a tender system. Suppliers who win the bids may have some difficulties that cause a delay in the delivery of the ordered medications.

“*I will say geographical location of the suppliers. If the headquarters is not in Kumasi, there is a delay in supply. And sometimes the quantities the suppliers have might not be enough, so these are some of the issues we face*” (*MS*).

Essentially, in a functional system, measures should be put in place to ensure continuity of supply through the setting of minimum ordering points, maximum ordering points, and emergency ordering points to guide ordering of medicines. There were means of getting around ordering challenges even though the process was not standardized.

“*Every morning we check the drugs we have so the ones where the quantities are down, we order* (*CP*).”

Expiration of medicines seemed to be a problem for all stakeholders who gave the problem a 5 rating. They all had measures put in place to curb expiries.

“*I will rate the difficulty at 5 and this depends on the product* (*DDPS*).”

“*We send list to prescribers telling them the drugs and when they will expire and sometimes, we call other hospitals and swap* (*CP*).”

Stakeholders emphasized the need for refresher courses on supply chain to help them remember what they had previously learnt and be abreast with new trends that are emerging.

“*More training will help from time to time so we learn new ways to do the job* (*MS*).”

### 3.9. Accessibility of Medicines: The Role of the NHIA

Patients on the NHIS can obtain covered medicines from both hospitals. Additionally, KATH offers alternative measures for its clients if they are unable to supply their clients' prescribed drugs. They reported

“*They write the drugs on NHIS prescription form, stamped, signed by the prescriber and given to the clients to obtain the drugs from NHIS-accredited pharmacies outside the facility* (*DDPS*).”

The reimbursement of the facilities by the NHIA for their supplied drug is mostly delayed, hence making availability of drugs at the facility irregular or reduced. It was also realized that the NHIS medicine list does not cover all antihypertensive medications; hence, clients are made to pay for those drugs. The delay in reimbursement of KATH by the NHIA poses a lot of challenges to the facility such as

“*Unavailability of drugs to clients resulting in the worsening of the disease condition of clients* (*MS*).”

“*There is also 10% deduction in the total amount owed KATH by NHIA to defray errors that occur in the NHIS claims forms which are not refunded to the facility when no errors are detected* (*DP*).”

## 4. Discussion

From our study, the majority of participants were aged over 50 years, and this is to be expected as the prevalence of hypertension is associated with increasing age; often, due to the development of arterial stiffness, a decrease in sensitivity to the baroreceptors as well as changes in the renal system results in alteration of sodium metabolism [21]. There were more females than males in the study, and this could partly be due to the fact that women seek health more often than men [22].

JNC 7 and 8 recommend the use of a calcium channel blocker or a diuretic as the first line in the management of hypertension in blacks [23]. The data obtained from the study were in line with this recommendation as a large number of participants were on a combination therapy with one of these antihypertensives or monotherapy with a diuretic or a calcium channel blocker, respectively. Comparatively, participants from KATH, a tertiary hospital, had more drug combinations as compared to UHS which is a secondary hospital. This confirms studies which assert that tertiary hospitals offer advanced care; patients usually present to tertiary facilities with complications, which may warrant use of multiple medicines [24].

A section of the participants had in addition to their regimen a beta blocker, hydralazine, or methyldopa which could partly be a result of the existence of resistant hypertension where BP is still greater than 140/90 even after the patient is given three or more antihypertensive drugs [25]. Statins, as part of the regimen for a section of the participants, were warranted for their beneficial effects of reducing the incidence of cardiovascular and cerebrovascular events in these patients [26]. ACEI/ARBs were prescribed in diabetics partly for the prevention or management of diabetic nephropathy.

Comparatively, a higher section of the participants at KATH could not recall information on their medications. This could partly be as a result of the number of antihypertensives given or inadequate education from health workers. Patients may receive inadequate information on their medicines due to the high workload in these hospitals. The former is in line with previous studies which have closely related forgetfulness to nonadherence and subsequent poor BP control [27]. There was a significant association between awareness of precautionary measures as well as counselling and BP control. Research has shown that 50% of patients who were given antihypertensives do not take the medicines as prescribed, and this can be partly associated with the lack of adequate information on their medicines, warranting the need to invest time in patient education as well as follow-ups to ensure patients are well informed on their condition as well as its management [28].

At both sites, blood pressure was uncontrolled in more than half of participants; however, control was poorer at KATH than at UHS. Uncontrolled blood pressure increases the likelihood of occurrence of cardiovascular-related morbidity and mortality. Therefore, as expected, some of the participants from both study sites presented with comorbidities such as diabetes, stroke, and chronic kidney diseases. This was in line with previous studies from sub-Saharan Africa which further recommended the need for BP control to prevent morbidity and mortality with resultant improvement in patients' quality of life [29].

From our study, the majority of patients from both facilities were not adherent to medications with UHS, reporting a comparatively better adherence rate than KATH. There was also a significant association between adherence and BP control. A rise in systolic BP of 20 mmHg and diastolic BP by 10 mmHg above 115/75 mmHg doubles the risk of the incidence of cardiovascular diseases, hence the urgency to maintain optimal BP levels [30]. Hypertensive patients should therefore be encouraged to adhere to therapy through constant education and be made to understand that adherence leads to good BP control and a positive impact on overall health. Studies have reported that common reasons for nonadherence included forgetting to take antihypertensives and incidence of adverse drug events [31]. To enhance adherence, studies have recommended the institution of behavioural interventions to enable patients remember to take their medication [32], coordination of dosing with daily routines such as meals [33], introduction to reminder charts or technology for setting reminders [34], and empowering patients to give feedback on how they feel and the impact the medicines have on their lives [35]. These are measures which have been proven to increase adherence among people living with chronic diseases such as hypertension in similar populations and will be beneficial for patients with hypertension in both UHS and KATH.

Regarding accessibility to the healthcare facility, time taken to access UHS was relatively shorter as compared to KATH. This is because UHS provides primary healthcare mainly to the university community and surrounding suburbs, whilst KATH, a tertiary hospital, is the main referral hospital in the Ashanti Region. Regardless, the majority of participants reported that transportation was affordable. This implies that though the distances required to travel to the healthcare facilities vary, both facilities are relatively accessible to patients. From our study, there was a significant association between time taken to access the facility and adherence in both hospitals, and this has been previously shown by research to be significantly associated with blood pressure control [36, 37] as easy accessibility to these facilities promotes prompt hospital visit for refills and also reports easily to the healthcare facility when they encounter challenges that may hinder adherence and optimum BP control.

Accessibility to antihypertensive agents has been shown to be several times associated with good BP control [38, 39], and this was confirmed with our study where a majority of patients with sufficiently controlled blood pressure obtained 70% or more of their medications from the hospital pharmacy. This is an indication that ease of access to antihypertensive medications is an essential factor in promoting adherence and subsequently BP control. A study conducted showed that patients with insurance had good BP control as compared to those without insurance [40]. Although the majority of participants were covered by the NHIS in both facilities, the percentage of antihypertensive medications obtained from the hospital pharmacy at KATH was relatively lower than that at UHS. A recent study concluded that the NHIS has had an overall positive impact in enhancing access to medicines [41]. However, healthcare facilities nationwide have reported delays in reimbursement as well as low reimbursement rates. Due to this, not all medicines are fully covered by the NHIS or are readily accessible to patients. This in effect creates gaps in sustainable supply of medicines, and this could partly explain why the majority of the participants could not obtain all their medicines from the hospital [41]. This assertion was further confirmed by stakeholders in the facilities as they gave a similar report concerning the irregularities of NHIS processes. As previously discussed, central to the problem of inaccessibility to medicines is related to reimbursement issues for NHIS providers which bother on policy and governance. Improving NHIS processes is essential in making medicines easily accessible.

Ghana is endowed with more than 5,000 community pharmacies, and for most Ghanaians, the community pharmacy is their first port of call to meet their health needs. Recently, the Pharmacy Council of Ghana abolished the 400-meter rule which only allowed pharmacies to be situated 400 meters apart. The sole aim of this decision was to enhance accessibility to pharmaceutical services. It is therefore not surprising that alternative sources of obtaining antihypertensive medicines by participants were the community pharmacies [42]. It was deduced from the study that hospitals relied on community pharmacies with NHIS accreditation to fill in prescriptions which were not readily available. Although this is partly a solution, few pharmacies seek accreditation because existing facilities face issues such as reimbursement delays, tedious claim filing processes, and the delay in adjusting health insurance medicine prices to reflect current market prices. This makes the NHIS unattractive to community pharmacies which may render medicines inaccessible especially to patients who cannot pay out of pocket [43].

In recent times, supply chain management has been an area of focus, especially in the public sectors. Even though the relevance of efficient logistics systems in achieving positive outcomes in healthcare has been widely discussed, several studies have reported high stockout rates of essential medicine, rendering medicines inaccessible in developing countries. Our study identified lapses in logistics management on the part of stakeholders mostly in forecasting, ordering, and losses to expiry. Stakeholders reported a need for constant training of the aforementioned areas as part of an interventional measure in their bid to enhance accessibility, and this has been proven to be effective by several authors [44]. Logistics management information systems can also be incorporated in standard practice as they have been shown to be beneficial in making correct interpretations and predictions through the provision of accurate data reporting, enhanced visibility, and promotion of good supply chain decisions with resultant reduction in stockouts and optimized health outcomes [45]. We assert that efficient supply chain systems which contribute to ease of access of antihypertensives will improve adherence to antihypertensive medications resulting in good BP control.

## 5. Conclusion

We studied the impact of accessibility to antihypertensives and adherence on blood pressure control as well as the role of the supply chain and NHIS in making medicines accessible. Comparatively, BP control was poorer at KATH than at UHS. Antihypertensives were more accessible at UHS than at KATH, and there was a significant correlation between accessibility and BP control. We found out that accessibility is largely driven by good supply chain practices and prompt payment of claims by the NHIA. Knowledge on medications was significantly higher at UHS than at KATH with a significant correlation to BP control. Adherence was significantly associated with BP control with participants at UHS being comparatively adherent than at KATH. It is imperative that antihypertensives are made accessible, and more education is given on the management of hypertension to improve adherence and BP control. Other factors affecting accessibility as well as other methods to improve adherence amongst hypertensives such as follow-ups and phone reminders can be studied in Ghana.

## Figures and Tables

**Table 1:** Demographic characteristics of participants.

Parameter	UHS (*n* = 143)	KATH (*n* = 342)	Distribution
*Age*			
30–40 years	4 (2.8%)	21 (6.1%)	0.225
41–50 years	25 (17.5%)	47 (13.7%)	
51–60 years	31 (21.7%)	88 (25.7%)	
61–70 years	56 (39.2%)	186 (54.4%)	
More than 70 years	27 (18.9%)	0	

*Sex*			
Male	48 (33.8%)	94 (27.5%)	−0.692
Female	94 (66.2%)	248 (72.5%)	

*Duration of the disease*			
1–5	42 (29.4%)	54 (15.8%)	0.230
6–10	66 (46.2%)	73 (21.3%)	
11–15	17 (11.9%)	118 (34.5%)	
16–20	15 (10.4%)	39 (11.4%)	
20 and above	3 (2.1%)	58 (17.0%)	

*Medication refill duration (months)*			
1–3	89 (62.1%)	118 (34.5%)	−0.532
4–6	54 (37.8%)	207 (60.5%)	
7–9	0	17 (5.0%)	

*BP categories (mmHg)*			
<120/80	21 (14.7%)	55 (16.1%)	−0.625
120–139/80–89	50 (34.9%)	106 (31.0%)	
Above 140/90	72 (50.4%)	181 (52.9%)	

*Comorbid conditions*			
Stroke	0	17 (5.0%)	0.332
CKD	7 (4.9%)	9 (2.6%)	
Diabetes	23 (16.1%)	9 (2.6%)	

**Table 2:** Accessibility to healthcare facilities.

Parameter	UHS (*n* = 143)	KATH (*n* = 342)	Distribution
*Time (minutes)*			
10–20	75 (53.2%)	35 (25.5%)	−1.34
21–30	36 (25.5%)	31 (22.6%)	
41–50	9 (6.4%)	46 (33.6%)	
51–60	21 (14.9%)	25 (18.2%)	
More than 60	0	205 (59.9%)	

*Mode of transport*			
Public transport	109 (76.2%)	245 (71.6%)	−1.18
Private transport	31 (21.7%)	84 (24.6%)	
Walking	3 (2.1%)	13 (3.8%)	

*Affordability of transportation*			
No	18 (1.8%)	13 (3.8%)	−1.32
Yes	125 (98.2%)	329 (96.2%)	

**Table 3:** Coverage of healthcare costs.

Parameter	UHS (*n* = 143)	KATH (*n* = 342)	Distribution
NHIS	124 (86.7%)	296 (86.5%)	−1.23
Private insurance	12 (8.4%)	1 (0.3%)	
Cash	5 (3.5%)	44 (12.9%)	

**Table 4:** Antihypertensive medications prescribed for participants.

Parameter	UHS (*n* = 143)	KATH (*n* = 342)
Medicines prescribed		
Calcium channel blocker + ACEI/ARB + diuretic (a)	45 (31.4%)	131 (38.2%)
Calcium channel blocker + ACEI/ARB (b)	49 (34.3%)	121 (35.4%)
Calcium channel blocker + diuretic	6 (4.2%)	37 (10.8%)
ACEI/ARB + diuretic (c)	8 (5.6%)	17 (4.9%)
Calcium channel blocker	17 (11.9%)	30 (8.8%)
Diuretic	3 (2.1%)	1 (0.3%)
Methyldopa	8 (5.6%)	101 (29.5%)
Hydralazine	0	37 (10.8%)
Statin in addition to the antihypertensive regimen	5 (3.5%)	106 (31.0%)
Beta blockers	2 (1.4%)	45 (13.1%)

a, b, and c are different combinations of antihypertensive medications used.

**Table 5:** Effect of knowledge and adherence on blood pressure control.

Parameter	UHS (*n* = 143)		KATH (*n* = 342)	
	Controlled	Uncontrolled	Controlled	Uncontrolled
Knowledge on precautionary measures				
No	24 (36.9%)	46 (59.0%)	37 (23.0%)	109 (60.2%)
Yes	41 (63.1%)	32 (41.0%)	124 (77.0%)	72 (39.8%)
Total	65 (100%)	78 (100%)	161 (100%)	181 (100%)
Adherence score				
Adherent (6–10)	37 (56.9%)	33 (42.3%)	94 (58.3%)	84 (46.4%)
Nonadherent (0–5)	28 (43.1%)	45 (57.7%)	67 (41.7%)	97 (53.6%)
Total	65 (100%)	78 (100%)	161 (100%)	181 (100%)

**Table 6:** Association between access to antihypertensive medications and blood pressure control.

Parameter	UHS (*n* = 143)		KATH (*n* = 342)	
	Controlled	Uncontrolled	Controlled	Uncontrolled
Percentages of medicines obtained from the hospital pharmacy				
70–100	46 (70.7%)	37 (47.4%)	98 (60.9%)	102 (56.4%)
69 and below	19 (29.3%)	41 (52.6%)	63 (39.1%)	79 (43.6%)
Total	65 (100%)	78 (100%)	161 (100%)	181 (100%)

## Data Availability

All the data obtained during this study are available from the corresponding author upon request.

## Supplementary Materials

Supplementary Materials A qualitative questionnaire was adapted from the WHO standard DTC handbook and the USAID supply chain KPI reference to assess stakeholders' commitment in making medicines readily available. A copy of the questionnaire has been included as supplementary materials.
