# Supplementary material for: Blood Pressure Control, Accessibility, and Adherence to Antihypertensive Medications: Patients Seeking Care in Two Hospitals in the Ashanti Region of Ghana
Source: Int J Hypertens. 2021 Jul 15;2021:9637760. doi: 10.1155/2021/9637760 (PMC8302388; doi:10.1155/2021/9637760)
Supplement: Supplementary Materials — A qualitative questionnaire was adapted from the WHO standard DTC handbook and the USAID supply chain KPI reference to assess stakeholders' commitment in making medicines readily available. A copy of the questionnaire has been included as supplementary materials. [file 9637760.f1.docx]

**Appendix**

**Questionnaires**

**Blood pressure control, accessibility, and adherence to antihypertensive medications: Patients seeking care in two hospitals in the Ashanti region of Ghana.**

Age: ……………. Gender ……………………… Weight …………………

NHIS ……………... BP ……………………... Date …………………

1. Where do you stay? …………………………………………………………………….
2. How many minutes/hours do you spend from your house to the hospital? …………………………………………
3. How do you get to the hospital? ………………………………………………………….
4. If you board a vehicle, are you able to afford the fare? …………………………………..
5. When were you diagnosed of hypertension? ………………………………………………
6. How long have you been on antihypertensive medications drugs? ……………………………………………
7. What are your anti-hypertensive drugs and how do you take them?

| Drug | | | Dosage | | | Duration | | |
| --- | --- | --- | --- | --- | --- | --- | --- | --- |
|  | Yes | No |  | Yes | No |  | Yes | No |
|  |  |  |  |  |  |  |  |  |
|  |  |  |  |  |  |  |  |  |
|  |  |  |  |  |  |  |  |  |

Co-morbidity…………………………………………………………………………………

1. Where do you get your hypertensive drugs from? A. Hospital pharmacy B. Community pharmacy C. Both hospital and community pharmacy D. Others
2. What percentage of your anti-hypertensive drugs do you often receive from prescribed facility?
3. 70-100% B. 40-60% C. Below 40%
4. How do you get your unavailable drugs? A. Hospital B. Community Pharmacy C. Both Hospital and community pharmacy D. Others ……………………………………….
5. How do you afford the cost of your anti-hypertensive drugs? A. NHIS B. Cash and Carry C. Private Health Insurance
6. How often do you come for reviews?
7. **Medication Adherence Rating Scale-10**

|  | **Question** | **Answer** |
| --- | --- | --- |
| 1 | Do you ever forget to take your medication? | Yes / No |
| 2 | Are you careless at times about taking your medication? | Yes / No |
| 3 | When you feel better, do you sometimes stop taking your medication? | Yes / No |
| 4 | Sometimes if you feel worse when you take the medication, do you stop taking it? | Yes / No |
| 5 | I take my medication only when I am sick | Yes / No |
| 6 | It is unnatural for my mind and body to be controlled by medication | Yes / No |
| 7 | My thoughts are clearer on medication | Yes / No |
| 8 | By staying on medication, I can prevent getting sick. | Yes / No |
| 9 | I feel weird, like a ‘zombie’ on medication | Yes / No |
| 10 | Medication makes me feel tired and sluggish | Yes / No |

1. Do you have some of your left-over medicines before you come for review? A. Yes B. No
2. Do you inform your doctor about the left-over medicine? A Yes B. No
3. If answer to Q. 15 is No, what do you do to the left-over medicines?

**Stakeholders’ Questionnaire**

**Accessibility of medicines; the role of supply chain**

1. Please describe your title, your years of work experience, and how long you have worked here.
2. How do you currently order medicines?
3. What are the issues you face during ordering of medicines?
4. What are the issues you face when you supply or dispense medicines?
5. What factors influence the quantities of medicines you order?
6. How do you know when to place an order for medicines?
7. How do you know what you will need for supplies in the future?
8. On a scale of 1-10, how difficult is it to decrease quantities lost to expiry? Please explain
9. What methods do you put in place to make sure that you don’t experience issues of drugs expiring?
10. What process do you follow currently when receiving new supplies?
11. Do you think you have everything you need now to be able to perform your job very well?
12. In general, what aspects of your job would you like to be able to do better?
13. What would help you to do your job better?

**Accessibility of medicines; the role of NHIA**

1. Does your facility provide NHIS services?
2. How often does the NHIS embers the facility?
3. What happens if NHIS defaults in re-imbursement to Kath?
4. How are hypertensive patients treated when NHIS defaults in their re-imbursement to your facility?
5. Are all the anti-hypertensive drugs on the NHIS scheme?
6. How costly are the anti-hypertensive drugs not on the NHIS scheme?
7. What are the challenges you encounter in the discharge of your duties?
8. Do you have a drug/medicine therapeutic committee?
9. When was the DTC/MTC formed?
10. How often do the D/MTC meet?
11. How many are the D/MTC members?
12. What is the composition of D/MTC members?
13. Do you have a drug formulary?
14. How did you formulate the drug formulary?
15. How often do you meet to review the drug formulary?
16. What is the percentage of tracer drug availability at the hospital for the past 12 months?
17. Do you have a hypertensive guideline/formula for use at the facility?
18. If yes, how often do you review both the hypertensive guidelines/formulary?
19. Where/how do you purchase your hypertensive drugs for the facility?
20. Are the purchased drugs cost effective?
21. If you do not get the drugs from your first source, what alternative source do you get your drugs from?
22. Are your purchased drugs of good quality and standardised?
23. If patients are not able to afford their prescribed hypertensive drugs, what alternative measures does the facility have for them?
